# Catecholaminergic manipulation alters dynamic network topology across cognitive states

**DOI:** 10.1162/netn_a_00042

**Published:** 2018-09-01

**Authors:** James M. Shine, Ruud L. van den Brink, Dennis Hernaus, Sander Nieuwenhuis, Russell A. Poldrack

**Affiliations:** Department of Psychology, Stanford University, Stanford, CA, USA; Brain and Mind Centre, The University of Sydney, Sydney, NSW, Australia; Institute of Psychology, Leiden University, Leiden, The Netherlands; Leiden Institute for Brain and Cognition, Leiden, The Netherlands; Maryland Psychiatric Research Center, University of Maryland School of Medicine, MD, USA; Institute of Psychology, Leiden University, Leiden, The Netherlands; Leiden Institute for Brain and Cognition, Leiden, The Netherlands; Department of Psychology, Stanford University, Stanford, CA, USA

**Keywords:** fMRI, Noradrenaline, Integration, Flexibility, Network, Atomoxetine

## Abstract

The human brain is able to flexibly adapt its information processing capacity to meet a variety of cognitive challenges. Recent evidence suggests that this flexibility is reflected in the dynamic reorganization of the functional connectome. The ascending catecholaminergic arousal systems of the brain are a plausible candidate mechanism for driving alterations in network architecture, enabling efficient deployment of cognitive resources when the environment demands them. We tested this hypothesis by analyzing both resting-state and task-based fMRI data following the administration of atomoxetine, a noradrenaline reuptake inhibitor, compared with placebo, in two separate human fMRI studies. Our results demonstrate that the manipulation of central catecholamine levels leads to a reorganization of the functional connectome in a manner that is sensitive to ongoing cognitive demands.

## Author Summary

There is emerging evidence that the flexible network structure of the brain is related to activity within the ascending arousal systems of the brain, such as the noradrenergic locus coeruleus. Here, we explored the role of catecholaminergic activity on network architecture by analyzing the graph structure of the brain measured using functional MRI following the administration of atomoxetine, a selective noradrenaline reuptake inhibitor. We estimated functional network topology in two double-blind, placebo-controlled datasets: one from the resting state and another from a parametric N-back task. Our results demonstrate that the nature of catecholaminergic network reconfiguration is differentially related to cognitive state and provide confirmatory evidence for the hypothesis that the functional network signature of the brain is sensitive to the ascending catecholaminergic arousal system.

## INTRODUCTION

A fundamental question facing modern neuroscience is how local computations are integrated across the brain to support the vast repertoire of mammalian behavior and cognition. Convergent results from multimodal neuroimaging studies (de Pasquale, Penna, Sporns, Romani, & Corbetta, 2016; Kitzbichler, Henson, Smith, Nathan, & Bullmore, 2011; Shine et al., 2016; Vatansever, Menon, Manktelow, Sahakian, & Stamatakis, 2015) have demonstrated that brain activity during cognitive tasks reflects a balance between regional segregation and network-level integration (Shine & Poldrack, in press), in which communication across distributed circuits enables fast and effective cognitive performance (Shine et al., 2016).

There is growing evidence that ascending catecholaminergic neuromodulatory projections from the brainstem mediate this integration (Samuels & Szabadi, 2008; Shine et al., 2016). Projections from arousal-related nuclei, such as the noradrenergic locus coeruleus (Sara, 2009), arborize widely in target regions and putatively alter network architecture by modulating the impact of incoming neuronal input in an activity-dependent manner (Aston-Jones & Cohen, 2005). Previous neuroimaging studies in humans have highlighted a close relationship between noradrenaline, network topology, and cognitive performance (Eldar, Cohen, & Niv, 2013; Shine et al., 2016). Specifically, increased free noradrenaline has been shown to increase the phasic-to-tonic ratio of neuronal firing in both the locus coeruleus and the cortex. As such, neurons that are less tonically active during the unstimulated state may also simultaneously demonstrate a heightened responsivity to relevant stimuli (Bari & Aston-Jones, 2013; Devilbiss & Waterhouse, 2011). We have previously used a biophysical computational model to demonstrate that fluctuations in neural gain, the potential computational role of catecholamines (Aston-Jones & Cohen, 2005; Servan-Schreiber, Printz, & Cohen, 1990), controls the balance between network-level segregation and integration (Shine, Aburn, Breakspear, & Poldrack, in press). However, it is not yet known whether directly manipulating noradrenaline shapes network topology, or indeed whether the effects of noradrenergic function on network topology differ across behavioral contexts.

To test the hypothesis that ascending catecholamines modulate global network topology as a function of cognitive state, we analyzed two separate fMRI datasets in which individuals were scanned following administration of either atomoxetine (ATX), a noradrenergic reuptake inhibitor (Robbins & Arnsten, 2009), or a pharmacologically inactive placebo. In the first study, subjects were scanned in the “resting” state (van den Brink et al., 2016); in the second, subjects were scanned while performing a cognitively challenging N-back task (Hernaus, Casales Santa, Offermann, & Van Amelsvoort, 2017). Based on the opposing effects of ATX on functional connectivity observed in these two studies (Hernaus et al., 2017; van den Brink et al., 2016), animal studies that highlight differential effects of ATX on phasic versus tonic locus coeruleus activity (Bari & Aston-Jones, 2013) and the hypothesized link between nor adrenaline and network topology (Eldar et al., 2013; Shine et al., 2016), we expected that ATX administration would manifest distinct topological effects as a function of cognitive state.

## RESULTS

### Effect of Atomoxetine on the Topological Signature of the Resting State

In the double-blind, placebo-controlled crossover resting-state study (van den Brink et al., 2016), 24 healthy subjects (age = 19–26) underwent fMRI scanning prior to (t = −20 minutes) and following (t = +90 minutes) the administration of either 40 mg of ATX or placebo. To estimate time-resolved network topology, we submitted preprocessed BOLD fMRI data from each subject to a preregistered analysis pipeline that calculates sliding-window connectivity between regional time series (Shine et al., 2015) (Supporting Information Figure S1a, Shine, van den Brink, Hernaus, Nieuwenhuis, & Poldrack, 2018) and then estimates the resulting topological signature of each windowed graph (Shine et al., 2016). Specifically, we used a weighted- and signed-version of the Louvain algorithm (Rubinov & Sporns, 2010) to identify tightly connected communities of regions within each temporal window. We then determined how strongly each region was connected to other regions within its own module (quantified using the within-module degree Z-score: W_*T*_) as well as to regions outside of its own module (quantified using the participation coefficient: B_*T*_) over time. The resultant topology can be summarized at the regional level (e.g., to determine which regions were the most integrated during a particular cognitive state) or at the global level by using a joint histogram of W_*T*_ and B_*T*_ values (known as a “cartographic profile”). Rightward fluctuations in the density of the cartographic profile along the horizontal (i.e., B_*T*_) axis reflect a more highly integrated functional connectome and have been shown to relate positively to individual differences in effective cognitive performance (Mattar, Cole, Thompson-Schill, & Bassett, 2015; Shine et al., 2016). Importantly, the participation coefficient measures the distribution of connections rather than their magnitude, such that B_*T*_ can be elevated in cases with relatively weak overall connectivity between regions.

**Figure 1.**
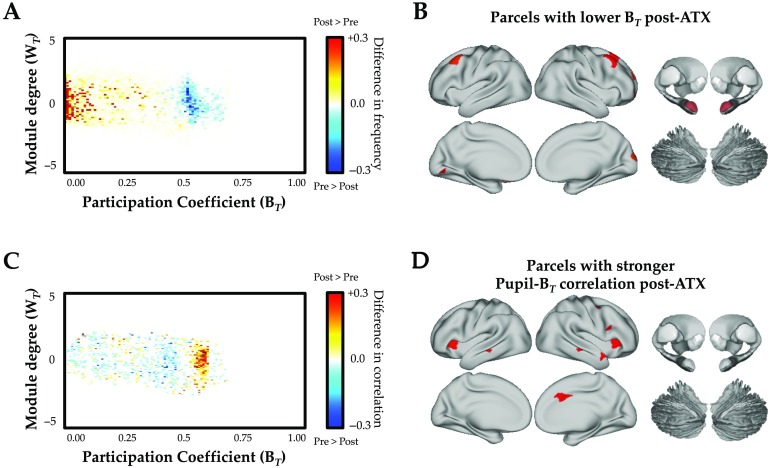
(A) Effect of atomoxetine versus placebo on the cartographic profile, which demonstrates a shift toward segregation: red/yellow, increased frequency postatomoxetine; and blue, decreased frequency postatomoxetine (FDR *q* ≤ 0.05). (B) Parcels with decreases in their between-module connectivity (i.e., participation coefficient) following atomoxetine (vs. placebo); see Supporting Information Table S1 (Shine et al., 2018) for parcel MNI coordinates (FDR *q* ≤ 0.05). (C) Effect of atomoxetine versus placebo on the relationship between the cartographic profile and pupil diameter, which demonstrates a shift toward integration: red/yellow, increased frequency postatomoxetine; and blue, decreased frequency postatomoxetine (FDR *q* ≤ 0.05). (D) Parcels with increased time-varying connectivity between between-module connectivity (i.e., participation coefficient) and pupil diameter following atomoxetine (vs. placebo); see Table S1 for parcel MNI coordinates (FDR *q* ≤ 0.05). Key: ATX, atomoxetine; B_*T*_, between-module connectivity; W_*T*_, within-module connectivity; see Table S1 for parcel coordinates.

As predicted (https://osf.io/utqq2), the administration of ATX compared with placebo at rest led to a significant reconfiguration of network-level topology (Figure 1A). Specifically, ATX administration caused a global shift toward segregation that was maximal in lateral frontal, frontopolar, and occipital cortices, along with the bilateral amygdala (Figure 1B). A parsimonious explanation for this result is that increases in free synaptic noradrenaline are known to downregulate tonic activity within the locus coeruleus, which has a dense expression of inhibitory α2-autoreceptors (Bari & Aston-Jones, 2013; Sara, 2009). As such, our results suggest that network topology in the resting state became segregated because of a reduction in the tonic firing rate of the locus coeruleus (Bari & Aston-Jones, 2013). This interpretation is consistent with recent computational models that demonstrate a strong link between the tonic firing rate of the locus coeruleus and functional signatures of brain network activity (Safaai, Neves, Eschenko, Logothetis, & Panzeri, 2015).

### Network Topology is Sensitive to Phasic and Tonic Catecholaminergic Levels

Although the topological signature observed in the resting state is consistent with a decrease in tonic noradrenaline, in vivo experiments in rodents have demonstrated that ATX administration also enhances phasic firing patterns in the locus coeruleus (Bari & Aston-Jones, 2013). This in turn should be expected to potentiate phasic noradrenergic responses and hence integrate the brain; however, this will happen only under conditions necessary to elicit phasic noradrenergic signaling, such as sensory salience (Nieuwenhuis, De Geus, & Aston-Jones, 2011) and acute stress (Hermans, van Marle, & Ossewaarde, 2011). Thus, in the context of an increase in free noradrenaline, we might expect that the strength of the relationship between network topology and phasic noradrenaline should increase following ATX administration. That is, the presence of extra noradrenaline should facilitate additional network reconfiguration as a function of behavioral requirements.

The lack of cognitive constraints during the resting state make it inherently difficult to directly test whether the predicted alterations in phasic catecholaminergic activity were indeed related to changes in network topology. Fortunately, we could interrogate this hypothesis by leveraging the relationship between the locus coeruleus and the descending sympathetic circuitry that controls pupil dilation (Nieuwenhuis et al., 2011), which in turn has been linked to cognitively relevant alterations in cortical arousal (Joshi, Li, Kalwani, & Gold, 2016; McGinley, David, & McCormick, 2015a; Reimer et al., 2016). In a previous study, we demonstrated a positive relationship between pupil diameter and fluctuations in network topology (Shine et al., 2016), suggesting that ascending neuromodulatory signals may facilitate network-level integration. In the current study, we hypothesized that the increase in free catecholamines following atomoxetine (Warren, van den Brink, Nieuwenhuis, & Bosch, 2017) should heighten this relationship, and hence lead to a stronger relationship between pupil diameter and network- and regional-level integration. Consistent with this hypothesis, we observed a stronger relationship between pupil diameter and network topology following ATX administration than following placebo (Figure 1C and 1D). Together, these results provide evidence to suggest that during quiescence, network topology is sensitive to both phasic and tonic patterns of ongoing noradrenergic activity.

### Effect of Atomoxetine on the Topological Signature of Cognitive Function

A potential benefit of increasing the concentration of free noradrenaline (Invernizzi & Garattini, 2004) is that the liberated catecholamines can be utilized in appropriate contexts to facilitate activity within task-relevant neural circuits. In other words, ATX may downregulate tonic noradrenergic release during rest, but when required, it may conversely facilitate an increase in phasic noradrenergic release (Bari & Aston-Jones, 2013) and hence increase network-level integration. To directly test this hypothesis, we analyzed data from a separate dataset of 19 subjects (age range 18–30) who underwent a cognitively challenging, parametric N-back task after the administration of either ATX (60 mg) or placebo (Hernaus et al., 2017) (Supporting Information Figure S1b, Shine et al., 2018). We hypothesized that because of a heightened phasic noradrenergic response, the main effect of network-level integration should be more pronounced during the task following ATX, when compared with the placebo condition.

**Figure 2.**
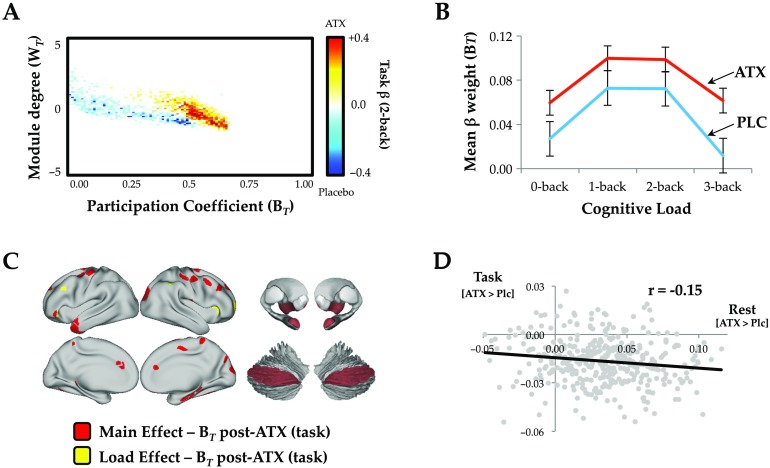
(A) Mean cartographic profile across all four blocks of load comparing post-ATX to postplacebo; similar patterns were observed in each block (FDR *q* ≤ 0.05). (B) Mean parcelwise B for each N-back load in both the placebo (PLC, blue) and atomoxetine (ATX, red) conditions (error bars represent standard error across subjects). (C) Parcels with higher B_*T*_ post-ATX as a function of task performance (FDR *q* ≤ 0.05); main effect (red) and load effect (yellow). (D) Correlation between the regions that showed highest B_*T*_ during task performance (ATX > Placebo) and regions that were shifted toward segregation in the rest study (ATX_(Post>Pre)_ > Placebo_(Post>Pre)_); see Supporting Information Table S1 (Shine et al., 2018) for parcel MNI coordinates (FDR *q* ≤ 0.05). Key: ATX, atomoxetine; B_*T*_, between-module connectivity; W_*T*_, within-module connectivity.

Consistent with our hypothesis, we observed a significant increase in network-level integration during task performance following ATX (Figure 2A). Specifically, there was an inverted U-shaped relationship between cognitive load and network integration in both conditions that was significantly elevated in the post-ATX session (t = 2.47; *p* = 0.009; Figure 2B). This main effect of ATX was maximal across frontal, parietal, and temporal cortices, along with thalamus, amygdala, and Crus II of the cerebellum (Figure 2C, red). Importantly, there is a long-standing research literature linking catecholamines with cognitive function via an inverse U-shaped relationship (Cools & D’Esposito, 2011; Robbins & Arnsten, 2009); however, few studies have provided a potential explanation for the algorithmic benefits that such a mechanism might confer. Here, we demonstrate that network integration may mirror the inverted U-shaped relationship between catecholamine levels and cognitive performance.

### Regional Topological Signatures Change as a Function of Cognitive Load

Although the majority of regions across the brain demonstrated an inverse U-shaped relationship with load, there was a subset of regions that demonstrated a linear increase with cognitive load following atomoxetine administration (Figure 2C, yellow). Specifically, the bilateral anterior insula, left dorsolateral prefrontal cortex, and right frontopolar cortex demonstrated a higher extent of integration (B_*T*_) with increasing task complexity following ATX, suggesting that the additional free catecholamines may have facilitated enhanced topological involvement of these regions as a function of task performance. Together, the relationship between these regions and ATX suggests that phasic noradrenaline may selectively enhance performance in task-relevant regions, perhaps through arousal-mediated alterations in neural gain (Devilbiss & Waterhouse, 2011; Reimer et al., 2014; Waterhouse, Moises, & Woodward, 1980).

### Differential Effects of Catecholamines on Network Topology as a Function of Cognitive State

The topological dissociation across the two studies analyzed suggests that the effect of ATX on network topology may be mediated by a set of similar regions across cognitive states. When we directly compared the effect of ATX in the two datasets, we observed a spatial correspondence between the effects of ATX on network topology during rest and task. Specifically, the regional topological signature observed during the N-back task was inversely correlated with the regional signature observed during rest (*r* = −0.147 [bootstrapped 95% CI: −0.246 to −0.037]; *p* < 0.002; Figure 2D), suggesting that ATX affected similar regions during rest and task, albeit by shifting them in different topological directions.

### Differential Effects of Catecholamines on Network Flexibility as a Function of Cognitive State

**Figure 3.**
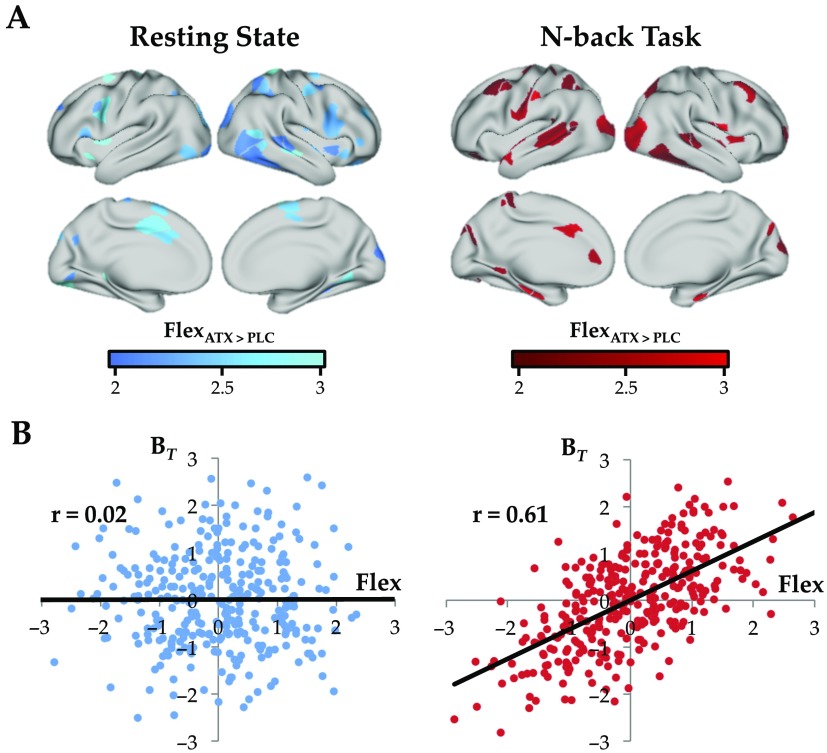
(A) Effect of atomoxetine (vs. placebo) on regional flexibility in the resting state (left, blue) and during the N-back task (right, red); regions depicted with increased flexibility (FDR *q* ≤ 0.05). (B) Correlation between effect of atomoxetine (vs. placebo) on B_*T*_ and regional flexibility during rest (left, blue; *r* = 0.02) and during the N-back task (right, red; *r* = 0.61); the difference between the two correlations was also significant (ZI* = 2.08; *p* = 0.037).

Previous work has shown that topological flexibility is important for cognitive performance (Cole et al., 2013; Medaglia, Lynall, & Bassett, 2015). That is, regions that are important for defining network architecture should both play a crucial role in network topology (e.g., interconnect otherwise disparate modules), while also maintaining temporal flexibility (e.g., so that network resources can be deployed at the appropriate time). To test whether atomoxetine administration exerted a significant effect on time-resolved network topology as a function of cognitive state, we calculated the topological flexibility of each region following atomoxetine (vs. placebo) under both task conditions. Specifically, we estimated the flexibility of each brain parcel by calculating the percentage of temporal windows in which an individual region “switched” between modules over the course of each session. We found that atomoxetine caused an increase in flexibility in a distributed network of regions across temporal, parietal, and frontal cortex, with distinct signatures in rest and task (Figure 3A). Interestingly, the administration of atomoxetine caused an increase in flexibility and integration in the same set of regions during the N-back task, but not during rest (ZI* = 2.08; *p* = 0.037; Figure 3B), suggesting that heightened free catecholamine levels may have facilitated network-level integration, but only during situations with tight cognitive constraints.

## DISCUSSION

Our results provide direct evidence that the manipulation of catecholamine levels in the human brain leads to substantial shifts in network topology. Furthermore, we were also able to demonstrate that the alterations in network topology critically depend on cognitive state. In the resting state, an overabundance of free catecholamine levels following ATX administration was associated with a relatively segregated network topology (Figure 1A). The lack of effortful cognitive engagement during the resting state may have facilitated a decrease in ascending arousal via ATX-mediated autoinhibition of the locus coeruleus (Bari & Aston-Jones, 2013; Sara, 2009), allowing the network to settle into a segregated architecture, potentially as a way to minimize energy expenditure (Bullmore & Sporns, 2012). In contrast, when presented with a complex cognitive challenge following ATX, an increase in the phasic-to-tonic ratio of noradrenergic function (Bari & Aston-Jones, 2013) may have facilitated functional connectivity between otherwise segregated circuits, integrating the functional connectome (Figure 2A) and putatively increasing the temporal coordination between the brain circuitry required to successfully complete the N-back task. Together, these results thus provide evidence that the ascending arousal system mediates the balance between network-level integration and segregation as a function of cognitive demands.

The administration of atomoxetine also differentially effected time-varying network topology as a function of cognitive state. During the resting state, atomoxetine affected switching and network integration relatively independently (Figure 3B). In contrast, the administration of atomoxetine during N-back task performance led to an increase in topological flexibility in the same regions that showed an increase in network-level integration (as measured by B_*T*_; Figure 3B). These results suggest that fluctuating levels of catecholaminergic neurotransmitters shape the spatiotemporal architecture of the brain in a manner that is sensitive to ongoing cognitive demands (Shine & Poldrack, in press). However, it bears mention that some of the observed differences between rest and task in our study may have been due to dose-related differences, particularly given the known differences in the sensitivity of different classes of noradrenergic receptors (Robbins & Arnsten, 2009). Irrespective of any potential dose-related effects, our results do provide empirical support for the hypothesis that fluctuations in noradrenaline are responsible for reconfiguring the network architecture of the brain. Indeed, the shift toward higher integration is consistent with the existing hypothesis that heightened phasic noradrenergic responses bias the network toward salient stimuli by modulating the sensitivity of the network to incoming sensory input (Sara & Bouret, 2012).

The precise biological mechanism underlying these effects is currently a topic of active investigation, but there is emerging evidence that the network-level impact of catecholamines may relate to their ability to modulate the “gain” of neurons across the brain (Aston-Jones & Cohen, 2005; McGinley et al., 2015b). For instance, it has previously been shown that stimulation of the locus coeruleus, both using electrical (Toussay, Basu, Lacoste, & Hamel, 2013) and optogenetic approaches (Carter et al., 2010), leads to the widespread activation of the cortex, producing a high-frequency, low-amplitude signature that is a known correlate of the awake brain (Berridge & Waterhouse, 2003). Similar patterns have also been observed during spontaneous activity in awake mice, confirming that firing in the locus coeruleus directly facilitates high-frequency cortical activity during natural behavior (Safaai et al., 2015). We recently used a biophysical computational model to show that manipulating neural gain transitioned the network from a segregated to an integrated architecture (Shine et al., 2018). The results of this study are consistent with these findings, and further confirm the role of catecholaminergic tone in simultaneously balancing the key topological properties of integration and segregation in a state-dependent manner. They also provide a mechanistic explanation for the brain’s response to periods of acute stress, which are also mediated by ascending noradrenergic systems (Hermans et al., 2011). Future studies will play an important role in solidifying this mechanistic explanation and determining the contexts in which the balance between these factors is most crucial for understanding complex behavior.

Although our experimental results suggest a crucial role for noradrenaline in the topological reconfiguration of brain network architecture, it bears mention that biological systems rarely demonstrate sharp boundaries between function systems. For instance, in addition to modulating noradrenaline, ATX administration has also been shown to modulate the central concentrations of other arousal-related neurotransmitters, including serotonin (Ding et al., 2014), histamine (Liu et al., 2008), and dopamine (Robbins & Arnsten, 2009), suggesting that the effects observed in our study may relate to the reconfiguration of the ascending arousal system as a whole. This systemic interdependence is perhaps best exemplified when comparing the relationship between noradrenaline and dopamine, the two major catecholaminergic neurotransmitters in the central nervous system. Whereas the majority of dopaminergic synapses utilize their own specific transporter (Chen & Reith, 2000), a subgroup of dopaminergic terminals in the cortex can also exploit noradrenergic transporters to reenter presynaptic axons (Morón, Brockington, & Wise, 2002). In addition, it has been shown that locus coeruleus neurons can corelease noradrenaline and dopamine (Devoto, Flore, Pira, & Longu, 2004). As such, our results may reflect the combined improvements in cortical signal-to-noise that relate to some combination of dopaminergic and noradrenergic effects on neuronal projection targets (Robbins & Arnsten, 2009). The different concentrations of ATX used in the two studies may also have affected these nonselective aspects of ATX. Fortunately, future studies that contrast the roles of the related neurotransmitter systems at different concentrations across a range of cognitive states will help to clarify this issue.

Together, our results demonstrate a relationship between network topology and catecholaminergic function that is sensitive to cognitive state. Future experiments should now be designed to decipher the relative impact of other neurotransmitter systems, both in health and disease.

## METHODS

### Resting-State Study

#### Participants.

Twenty-four right-handed individuals (age 19–26 years; 5 men) were included in this study. All participants were screened by a physician for physical health and drug contraindications. Exclusion criteria included the following: standard contraindications for MRI; current use of psychoactive or cardiovascular medication; a history of psychiatric illness or head trauma; cardiovascular disease; renal failure; hepatic insufficiency; glaucoma; hypertension; drug or alcohol abuse; learning disabilities; poor eyesight; smoking >5 cigarettes a day; and current pregnancy. All participants gave written, informed consent before the experiment.

#### Study design.

We used a double-blind placebo-controlled crossover design (van den Brink et al., 2016). In each of two sessions, scheduled 1 week apart at the same time of day, participants received either a single oral dose of atomoxetine (40 mg) or placebo (125 mg of lactose monohydrate with 1% magnesium stearate, visually identical to the drug). In both sessions, participants were scanned once before pill ingestion (t = −20 min) and once following ingestion (t = 90 min), when approximate peak plasma levels were reached. Each scan comprised 8 min of eyes-open resting-state fMRI. During scanning, the room was dark, and participants fixated on a black fixation cross presented on a gray background. Drug uptake was confirmed using cortisol and α-amylase levels in the saliva (van den Brink et al., 2016; Warren et al., 2017).

#### MRI data.

All MRI data were collected with a Philips 3T MRI scanner. In each of the scanning sessions, we collected T2*-weighted EPI resting-state images (echo time 30 ms, repetition time 2.2 s, flip angle 80°, FOV 80 × 80 × 38 voxels of size 2.75 mm isotropic, and 216 volumes). To allow magnetic equilibrium to be reached, the first five volumes were automatically discarded. In addition, each time the participant entered the scanner, we collected a b0 field inhomogeneity scan (echo time 3.2 ms, repetition time 200 ms, flip angle 30°, and FOV 256 × 256 × 80 voxels with a reconstructed size of 0.86 × 0.86 mm with 3-mm-thick slices). Finally, at the start of the first session, we collected a high-resolution anatomical T1 image (echo time 4.6 ms, repetition time 9.77 ms, flip angle 8°, and FOV 256 × 256 × 140 voxels with size 0.88 × 0.88 mm with 1.2-mm-thick slices) for image normalization and registration.

#### Data preprocessing.

After realignment (using FSL’s MCFLIRT) and skull stripping (using BET), b0 unwarping was used to control for potential differences in head position across sessions. The b0 scans were first reconstructed into an unwrapped phase angle and magnitude image. The phase image was then converted to units of radians per second and median filtered, and the magnitude image was skull-stripped. We then used FEAT to unwarp the EPI images in the y-direction with a 10% signal loss threshold and an effective echo spacing of 0.333. The unwarped EPI images were then prewhitened, smoothed at 5 mm FWHM, and coregistered with the anatomical T1 to 2-mm isotropic MNI space (degrees of freedom: EPI to T1, 3; T1/EPI to MNI, 12). FMRIB’s ICA-based X-noiseifier (Salimi-Khorshidi, Douaud, & Beckmann, 2014) was used with pretrained weights to de-noise the imaging data.

Temporal artifacts were identified in each dataset by calculating framewise displacement (FD) from the derivatives of the six rigid-body realignment parameters estimated during standard volume realignment (Power et al., 2014), as well as the root-mean-square change in BOLD signal from volume to volume (DVARS). Frames associated with FD > 0.25 mm or DVARS > 2.5% were identified; however, as no participants were identified with greater than 10% of the resting time points exceeding these values, no trials were excluded from further analysis. There were no differences in head motion parameters between the four sessions (*p* > 0.500). Following artifact detection, nuisance covariates associated with the six linear head movement parameters (and their temporal derivatives), DVARS, physiological regressors (created using the RETROICOR method), and anatomical masks from the CSF and deep cerebral WM were regressed from the data by using the CompCor strategy (Behzadi, Restom, Liau, & Liu, 2007). Finally, in keeping with previous time-resolved connectivity experiments (Bassett, Yang, Wymbs, & Grafton, 2015), a temporal band-pass filter (0.01 < f < 0.125 Hz) was applied to the data. In addition, all results were replicable both with and without frame censoring at the predefined levels, as well as with and without global signal regression.

#### Brain parcellation.

Following preprocessing, the mean time series was extracted from 375 predefined regions-of-interest (ROIs). To ensure whole-brain coverage, we extracted the following: 333 cortical parcels (161 and 162 regions from the left and right hemispheres, respectively) by using the Gordon atlas (Gordon et al., 2014), 14 subcortical regions from Harvard-Oxford subcortical atlas (bilateral thalamus, caudate, putamen, ventral striatum, globus pallidus, amygdala, and hippocampus; http://fsl.fmrib.ox.ac.uk/), and 28 cerebellar regions from the SUIT atlas (Diedrichsen et al., 2009) for each participant in the study.

#### Time-resolved functional connectivity.

To estimate time-resolved functional connectivity (Hutchison et al., 2013; Sakoğlu et al., 2010) between the 375 ROIs, we used the multiplication of temporal derivatives approach (MTD; http://github.com/macshine/coupling/; Shine et al., 2015). The MTD is computed by calculating the pointwise product of temporal derivative of pairwise time series (Equation 1). To reduce the contamination of high-frequency noise in the time-resolved connectivity data, the MTD is averaged by calculating the mean value over a temporal window, *w*. Time-resolved functional connectivity was calculated between all 375 brain regions by using the MTD within a sliding temporal window of 15 time points (33 seconds at 2.2 s per window), which allowed for estimates of signals amplified at approximately 0.1 Hz. Individual functional connectivity matrices were then calculated within each temporal window, thus generating an unthresholded (i.e., signed and weighted) three-dimensional adjacency matrix (region × region × time) for each participant. The MTD for the pairwise interaction between region *i* and *j* is defined according to Equation 1:

$${MTD}_{ijt}=\frac{1}{w}\sum_{t-w/2}^{t+w/2}\frac{\left({dt}_{it}\times {dt}_{jt}\right)}{\left(\sigma_{{dt}_{i}}\times \sigma_{{dt}_{j}}\right)}$$ (1)

where *dt* is the first temporal derivative of the *i*^th^ or *j*^th^ time series at time *t*, *σ* is the standard deviation of the temporal derivative time series for region *i* or *j*, and *w* is the window length of the simple moving average. This equation can then be calculated over the course of a time series to obtain an estimate of time-resolved connectivity between pairs of regions.

#### Time-resolved community structure.

The Louvain modularity algorithm from the Brain Connectivity Toolbox (Rubinov & Sporns, 2010) was used to estimate both time-averaged and time-resolved community structure. The Louvain algorithm iteratively maximizes the modularity statistic, *Q*, for different community assignments until the maximum possible score of *Q* has been obtained (Equation 2). The modularity estimate for a given network is therefore a quantification of the extent to which the network may be subdivided into communities with stronger within-module than between-module connections.

$$Q_{T}=\frac{1}{v^{+}}\sum_{ij}\left(w_{ij}^{+}-e_{ij}^{+}\right)\delta_{M_{i}M_{j}}-\frac{1}{v^{+}+v^{-}}\sum_{ij}\left(w_{ij}^{-}-e_{ij}^{-}\right)\delta_{M_{i}M_{j}}$$ (2)

Equation 2 gives the Louvain modularity algorithm, where *v* is the total weight of the network (sum of all negative and positive connections), *w*_*ij*_ is the weighted and signed connection between regions *i* and *j*, *e*_*ij*_ is the strength of a connection divided by the total weight of the network, and *δ*_*MiMj*_ is set to 1 when regions are in the same community and 0 otherwise. Superscripts ^+^ and ^−^ denote all positive and negative connections, respectively.

For each temporal window, the community assignment for each region was assessed 500 times, and a consensus partition was identified using a fine-tuning algorithm from the Brain Connectivity Toolbox (http://www.brain-connectivity-toolbox.net/). This then afforded an estimate of both the time-resolved modularity (Q_*T*_) and cluster assignment (Ci_*T*_) within each temporal window for each participant in the study. To define an appropriate value for the *γ* parameter, we iterated the Louvain algorithm across a range of values (0.5–2.5 in steps of 0.1) for 100 iterations of a single subjects’ time-averaged connectivity matrix and then estimated the similarity of the resultant partitions by using mutual information. Across the cohort there were on average 3.9 ± 1.2 communities found in each window. A γ parameter of 1.1 provided the most robust estimates of topology across these iterations, both at the group and individual subject level. Alternatively, a multilayer implementation of the Louvain algorithm could be used, although this would require the tuning of a separate parameter, ω, that defines the strength of connection between layers (Mucha, Richardson, Macon, Porter, & Onnela, 2010).

#### Cartographic profiling.

Based on time-resolved community assignments, we estimated within-module connectivity by calculating the time-resolved module degree Z-score (W_*T*_; within-module strength) for each region in our analysis (Equation 3) (Guimerà & Nunes Amaral, 2005).

$$W_{iT}=\frac{\kappa_{iT}-{\overset{´}{\kappa }}_{s_{iT}}}{s_{\kappa_{s_{iT}}}}$$ (3)

Equation 3 gives the module degree Z-score, W_*iT*_, where κ_iT_ is the strength of the connections of region *i* to other regions in its module s_i_ at time $T,{\overset{´}{\kappa }}_{s_{iT}}$ is the average of κ over all the regions in s_*i*_ at time *T*, and $\sigma_{\kappa_{s_{iT}}}$ is the standard deviation of κ in s_i_ at time *T*.

To estimate between-module connectivity (B_*T*_), we used the participation coefficient, B_*T*_, which quantifies the extent to which a region connects across all modules (i.e., between-module strength):

$$B_{iT}=1-\sum_{s=1}^{n_{M}}{\left(\frac{\kappa_{isT}}{\kappa_{iT}}\right)}^{2}$$ (4)

Equation 4 gives the participation coefficient B_*iT*_, where κ_isT_ is the strength of the positive connections of region *i* to regions in module *s* at time *T*, and κ_iT_ is the sum of strengths of all positive connections of region *i* at time *T*. The participation coefficient of a region is therefore close to 1 if its connections are uniformly distributed among all the modules and 0 if all of its links are within its own module.

To track fluctuations in cartography over time, for each temporal window, we computed a joint histogram of within- and between-module connectivity measures, which we refer to here as a “cartographic profile” (Figure 1A). Code for this analysis is freely available at https://github.com/macshine/integration/.

#### Pupilometry.

Pupil size was measured from the right eye at 500 Hz with an MRI-compatible Eyelink 1000 eye tracker. Blinks and other artifacts were interpolated offline by using shape-preserving piecewise cubic interpolation. Pupil data were low-pass filtered at 5 Hz to remove high-frequency noise and Z-scored across conditions. Five participants were excluded from pupil-related analyses because of poor signal quality (≥50% of continuous time series interpolated) or missing data. Of the remaining participants, on average 20% ± 9% of the data were interpolated. We replicated our previous observation (Shine et al., 2016) of a positive relationship between pupil diameter and integrated network topology (Supporting Information Figure S2a, Shine et al., 2018).

#### Null model creation.

To determine whether the integrative signature of the brain was more dynamic than predicted by a stationary null model (Supporting Information Figure S2b, Shine et al., 2018; Laumann, Snyder, Mitra, & Gordon, 2016); surrogate data was created using a stationary Vector Auto Regressive model (order was set at 6 to match the expected temporal signature of the BOLD response in 2.2 s TR data). The mean covariance matrix across the entire experiment was used to generate 2,500 independent null datasets, which allows for the appropriate estimation of the tails of nonparametric distributions (Nichols & Holmes, 2002). These time series were then preprocessed using the same approach outlined for the BOLD data. For each analysis, we estimated the kurtosis of the mean B_*T*_ time series for each of the 2,500 simulations. We then calculated the 95th percentile of this distribution and used this value to determine whether the resting-state data fluctuated more frequently than the null model. We found that the dynamic network structure within the fMRI data had a higher kurtosis than the 95th percentile of the stationary null model (Supporting Information Figure S2b, Shine et al., 2018), suggesting the presence of time-varying patterns in the brain. However, this interpretation requires some caution, given that stationary null models are often unable to determine whether interesting time-varying changes are indeed occurring in time series data (Liégeois et al., 2017; Miller et al., 2017).

#### Statistical analyses.

The following hypotheses were preregistered with the Open Science Framework (https://osf.io/utqq2/):

*Hypothesis 1:* To explicitly test whether the resting brain fluctuates more frequently than a stationary null model, we calculated the kurtosis of the window-to-window difference in the mean B_*T*_ score for each iteration of a vector autoregression (VAR) null model (model order = 6). The mean covariance matrix across all 24 subjects from the preplacebo session was be used to generate 2,500 independent null datasets, which allows for the appropriate estimation of the tails of nonparametric distributions (Nichols & Holmes, 2002). These time series was then be filtered in a similar fashion to the BOLD data. For each analysis, we created a statistic for each independent simulation that summarized the extent of fluctuations in the null dataset. We then calculated the 95th percentile of this distribution and used this value to determine whether the resting-state data fluctuated more frequently than the null model (i.e., whether there deviations as extreme as the 95th percentile of the null dataset occur more than 5% of the time).

*Hypothesis 2:* We estimated the Spearman’s rho correlation between the convolved pupil diameter and the time series of each bin of the cartographic profile. We then fitted a linear mixed-effects model with random intercept to determine whether the correlation between each bin of the cartographic profile was more extreme than chance levels (FDR *q* ≤ 0.05).

*Hypothesis 3:* Group level differences were investigated by comparing each bin of the cartographic profile for all subjects prior to and postatomoxetine administration, as well as prior to and postplacebo using a 2 × 2 ANOVA design. Specifically, the percentage of time that each bin of the cartographic profile was occupied during the resting state for each of the four sessions was be entered into a 2 × 2 ANOVA. Our hypothesis predicted a significant interaction effect between pre- and postplacebo and pre- and postatomoxetine administration. We corrected for multiple comparisons by using a false discovery rate of *q* ≤ 0.05. Similar interaction effects were assessed at the regional level (i.e., regional topological measures, such as participation coefficient) by using a series of 2 × 2 ANOVA designs.

### Task-Based Study

Based on our interim results, we hypothesized that if phasic and tonic noradrenaline release differentially alter the balance between integration and segregation, then integration should be stronger following ATX during cognitive task performance. This hypothesis was not identified as part of our preregistration, but arose as a post hoc interrogation of the data. To test this hypothesis, we analyzed data from a different dataset, in which 19 participants (age 18–30, all right-handed males) underwent a cognitively challenging N-back task following either ATX (60 mg) or placebo in a double-blind, randomized placebo-controlled crossover design (PLC-ATX *n* = 8; ATX-PLC *n* = 11; Hernaus et al., 2017). The study was carried out in accordance with the Declaration of Helsinki and was approved by the local medical ethics committee of Maastricht University Medical Centre (NL53913.068.15). All participants gave written, informed consent prior to each session and were reimbursed for participation.

#### Cognitive task.

Participants performed a parametrically modulated N-back task, in which they were required to identify target letters that were presented for 1,000 ms on an LCD screen during MRI scanning. Targets consisted of letters that were the same as the letter presented one, two, or three trials previously (i.e., 1-back, 2-back, or 3-back, respectively). A further control condition was also involved, in which participants were asked to detect the letter ‘X’ (i.e., 0-back). Every task condition was presented three times in pseudorandom order (3–4 targets per block). Participants responded to targets and distractors with right index and middle finger button presses, respectively. Task effects were modeled for fMRI analysis by using a block design.

#### MRI data and preprocessing.

All MRI data were collected with a Siemens 3T MRI scanner. In each of the scanning sessions, we collected T2*-weighted EPI images (echo time 30 ms, repetition time 2.0 s, flip angle 77°, and 286 volumes). To allow magnetic equilibrium to be reached, the first five volumes were automatically discarded. In addition, each time the participant entered the scanner, we collected a b0 field inhomogeneity scan (echo time 3.2 ms, repetition time 200 ms, flip angle 30°, and FOV 256 × 256 × 80 voxels with a reconstructed size of 0.86 × 0.86 mm with 3-mm-thick slices). Finally, at the start of the first session, we collected a high-resolution anatomical T1 image (echo time 4.6 ms, repetition time 9.77 ms, flip angle 8°, and FOV 256 × 256 × 140 voxels with size 0.88 × 0.88 mm with 1.2-mm-thick slices) for image normalization and registration. Data were preprocessed in a similar fashion to the resting-state analysis, albeit without correction for physiological parameters, which were not collected in the study.

#### fMRI analysis.

Preprocessed BOLD data were subjected to the same time-resolved network analysis pipeline as to the one utilized for the resting-state analysis. Following this step, both regional (W_*T*_ and B_*T*_) and global (cartographic profile) time series were modeled against the blocks of the 0-, 1-, 2-, and 3-back conditions in both the post-ATX and postplacebo sessions. Instruction screens, rest blocks, and head motion parameters (6 linear parameters and their temporal derivatives) were also modeled. We then statistically compared the resultant β weights for each of the blocks separately using a series of *F* tests (FDR *q* ≤ 0.05) with the following two contrasts: i) main effects, which were modeled as the mean activity in the 1-, 2-, and 3-back blocks versus the 0-back block; and ii) load effects, which were modeled as a parametric increase in activity as a function of cognitive load across the four blocks. None of the effects were significantly correlated with head motion, either within- or between-subjects (*p* > 0.5).

Finally, we correlated the β weights for the main effect of ATX > PLC during the task for each parcel with the interaction effect of ATX_[Post>Pre]_ > PLC_[Post>Pre]_ on resting-state topology by using a Pearson’s correlation (the task data did not contain a “predrug” condition). The significance of this correlation was determined by randomly permuting the task-based effects 5,000 times and then reestimating the Pearson’s correlation between the shuffled effects and the original regional effects in the resting state. The inverse correlation between the two parcel values was more extreme than the 0.2nd percentile of the null distribution (i.e., *p* < 0.002). A nonparametric bootstrapping approach was also used to estimate a 95% confidence interval for the correlation (1,000 iterations).

#### Regional flexibility.

We estimated the flexibility of each brain parcel by calculating the percentage of temporal windows in which an individual region “switched” between modules, normalized to the total number of modules in the data (as estimated in the previous step). Code was obtained directly from the original author (http://www.danisbassett.com/resources/). As the modular assignment was essentially arbitrary within each unique temporal window, we used a version of the Hungarian algorithm (Kuhn, 1955), which is a combinatorial optimization algorithm that calculates the most efficient path between different layers of a multislice network, to assign regions to modules with consistent values over time. Regional flexibility values were then compared across atomoxetine and placebo trials. Pearson’s correlations were later used to compare the regional signature of flexibility with regional B_*T*_ values, and the Dunn and Clark statistic was used to compare the correlations between rest and task in both datasets (Bayer, 2016).

## AUTHOR CONTRIBUTIONS

James Shine: Conceptualization; Data curation; Formal analysis; Investigation; Methodology; Visualization. Rudy van den Brink: Data curation; Investigation; Methodology. Sander Niewenhuis: Data curation; Investigation; Methodology. Russell Poldrack: Conceptualization; Methodology; Visualization.

## FUNDING INFORMATION

James Shine was supported by an NHMRC CJ Martin Fellowship (GNT1072403).

## TECHNICAL TERMS

Segregation: A state in which there is a relatively high modularity in a network, i.e., there are distinct modules with weak interconnections.
Integration: A state in which there is a relatively low modularity in a network, i.e., there are less distinct modules with strong interconnections.
Network: A system comprising a set of distinct nodes, interconnected by a set of edges.
Noradrenaline: A catecholaminergic neurotransmitter that provides a widespread, modulatory role over neuronal processing in the central nervous system.
Atomoxetine: A chemical that inhibits the transporter chemical that normally facilitates the reuptake of noradrenaline.
Module: A group of nodes that is more tightly interconnected than the connections between modules.
Flexibility: The proportion of time that an individual node switches modules over time
