# Supplementary material for: Catecholaminergic manipulation alters dynamic network topology across cognitive states
Source: Netw Neurosci. 2018 Sep 1;2(3):381–96. doi: 10.1162/netn_a_00042 (PMC6145851; doi:10.1162/netn_a_00042)
Supplement: Supplementary file 1 [file netn-02-381-s001.pdf]

Shine, J.M., van den Brink, R.L., Hernaus, D., Nieuwenhuis, S., & Poldrack, R.A. (2018). Supporting Information "Catecholaminergic Manipulation Alters Dynamic Network Topology Across Cognitive States." *Network Neuroscience, 2(3),* 381-396. https://doi.org/10.1162/netn_a_00042

Region	X	Y	Z
$ATX_{Post\text{-}Pre}$ > $Plc_{Post\text{-}Pre}$ (Rest)			
Left Occipital (138)	-9	-78	-4
Left Superior Frontal (157)	-42	16	48
Right Superior Frontal (200)	22	21	46
Right Occipital (307)	14	-74	-2
Right Frontal Pole (317)	24	51	24
Right Frontal Pole (320)	31	52	10
Right Superior Frontal (327)	42	20	48
Left Amygdala (339)	-22	-10	-12
Right Amygdala (346)	22	-8	-12
Pupil Diameter and Integration (Rest)			
Left Anterior Insula (80)	-40	24	-14
Left Superior Temporal (127)	-54	-4	-10
Right Dorsal Anterior Cingulate (188)	4	22	28
Right Superior Temporal (225)	60	-24	-6
Right Anterior Insula (243)	28	18	-20
Right Dorsal Prefrontal (276)	40	18	28
Right Temporal Pole (290)	60	-10	-14
Main Effect (N-back)			
Left Dorsal Anterior Cingulate (28)	-9	25	28
Left Dorsal Anterior Cingulate (29)	-10	34	22
Left Medial Parietal (30)	-11	-48	60
Left Premotor (41)	-27	-7	46
Left Motor (45)	-37	-23	62
Left Premotor (48)	-17	-9	62
Left Frontal Operculum (79)	-47	39	-9
Left Temporal Pole (129)	-45	9	-37
Left Superior Frontal (145)	-16	49	37
Left Temporal Pole (159)	-51	7	-20
Right Dorsal Occipital (166)	22	-85	24
Right Dorsal Anterior Cingulate (183)	8	35	23
Right Medial Sensory (193)	12	-41	67
Right Medial Motor (195)	7	-8	51
Right Motor (197)	42	-11	47
Right Premotor (205)	29	-14	64
Right Premotor (207)	21	-6	65
Right Sensory (217)	49	-26	52
Right Dorsal Occipital (255)	18	-78	34
Right Middle Frontal (272)	38	29	36
Right Occipital (307)	14	-92	15
Right Ventral Temporal (312)	20	-11	25
Right Ventral Temporal (314)	30	-19	-19
Right Superior Frontal (316)	21	43	35
Right Middle Frontal (328)	39	10	43
Left Thalamus (334)	-8	-18	2
Right Thalamus (341)	8	-18	2
Left Crus II (358)	-24	-78	-40
Right Crus II (360)	24	-78	-40
Load Effect (N-back)			
Left Anterior Insula (85)	-44	33	-7
Left Dorsolateral Prefrontal (108)	-43	19	34
Right Parietal Operculum (167)	48	-43	42
Right Anterior Insula (249)	34	24	10
Right Frontal Pole (319)	24	59	5

Table S1 – MNI Co-ordinates for parcels associated with significantly increased integration (B_T) in each of the analyses presented in the manuscript (parcel number in parentheses).

Shine, J.M., van den Brink, R.L., Hernaus, D., Nieuwenhuis, S., & Poldrack, R.A. (2018). Supporting Information "Catecholaminergic Manipulation Alters Dynamic Network Topology Across Cognitive States." *Network Neuroscience*. Advance publication. https://doi.org/10.1162/netn_a_00042

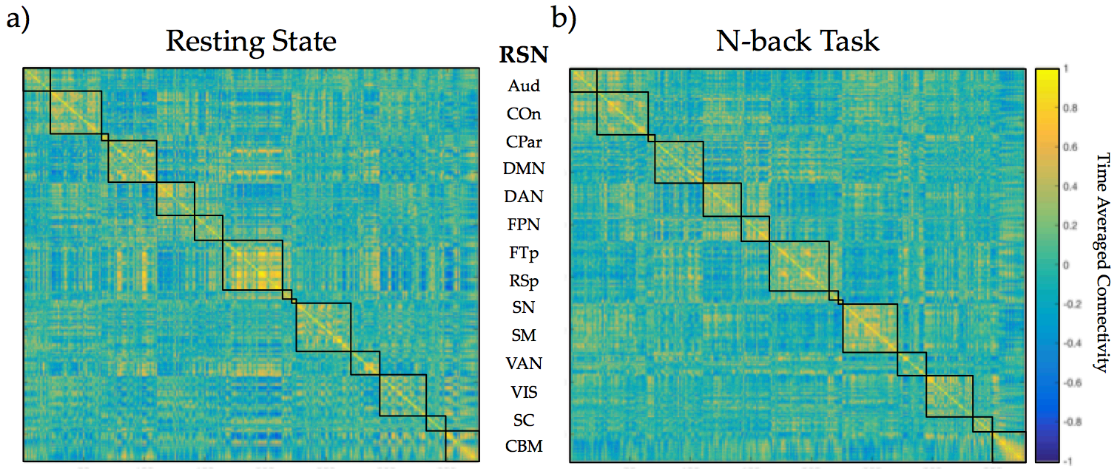

Figure S1 – Time-averaged connectivity matrix for resting state (a) and task (b), ordered by resting state network (RSN). Key: Aud – auditory; COn – cingulo-opercular; CPar – cingulo-parietal; DMN – default mode; DAN – dorsal attention; FPN – frontoparietal; FTp – frontotemporal; RSp – Retrosplenial; SN – salience; SM – somatomotor; VAN – ventral attention; VIS – visual; SC – subcortical; CBM – cerebellum.

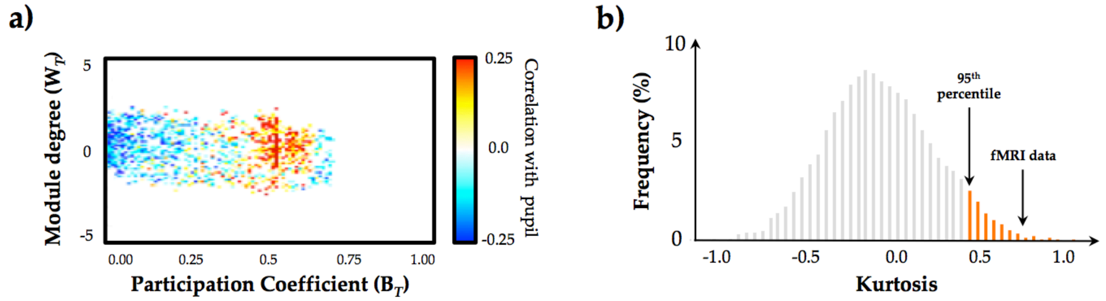

Figure S2 – a) cartographic profile demonstrating the group mean Pearson's correlation between pupil diameter and network-level integration across all four conditions; and b) a histogram showing results of a permutation analysis. The results demonstrate that the data used in our experiment displayed higher kurtosis (indicative of non-stationarity) than the results of 5000 iterations of a stationary vector autoregressive null model that was applied to the same methodological pipeline as the fMRI data from our experiment.
